# The association between Geriatric Nutritional Risk Index and the risk of Invasive Candidiasis in critically ill older adults

**DOI:** 10.1186/s12879-023-08512-5

**Published:** 2023-08-14

**Authors:** Yongqiang Dong, Heqing Tao, Ligang Liu, Ziyan Ni, Zhandong Yang, Kequan Chen, Shuying He, Liang Peng, Xueqing Chen

**Affiliations:** 1 Deartment of Thyroid Surgery, First Affiliated Hospital of Zhengzhou University, Zhengzhou, P.R. China; 2 Department of Gastroenterology, The First Affiliated Hospital of Guangzhou Medical University, Guangzhou Medical University, Guangzhou, 510120 P.R. China; 3 Institute of Therapeutic Innovations and Outcomes, College of Pharmacy, The Ohio State University, Columbus, USA

**Keywords:** Geriatric nutritional risk index, Invasive candidiasis, Propensity score match, Critically ill

## Abstract

**Background:**

Invasive candidiasis is the most common hospital-acquired fungal infection in intensive care units (ICU). The Geriatric Nutritional Risk Index (GNRI) score was developed to evaluate the nutritional status of elderly adults. We aimed to assess the association between the GNRI score and the risk of invasive candidiasis in elderly patients admitted to ICU.

**Methods:**

Hospitalization information of elderly patients with invasive candidiasis was collected retrospectively from Medical Information Mart for Intensive Care (MIMIC) IV and MIMIC-III Clinical Database CareVue subset from 2001 to 2019. The main outcome of this study was the diagnosis of invasive candidiasis in patients. We employed a multivariable Cox regression and propensity score matching to balance the influence of confounding factors on the outcome. Furthermore, we conducted sensitivity analyses by categorizing the GNRI into classes based on thresholds of 98, 92, and 81.

**Results:**

A total of 6739 patients were included in the study, among whom 134 individuals (2%) were diagnosed with invasive candidiasis. The GNRI scores of patients with invasive candidiasis upon admission to the ICU were significantly lower, measuring 88.67 [79.26–98.27], compared to the control group with a score of 99.36 [87.98-110.45] (P < 0.001). The results of the multivariable Cox regression analysis demonstrated a strong association between higher GNRI scores and a decreased risk of invasive candidiasis infection (HR: 0.98, 95% CI: 0.97–0.99, P = 0.002). Consistently, similar results were obtained when analyzing the propensity score-matched cohort (HR: 0.99, 95% CI: 0.98-1, P = 0.028). Sensitivity analyses further confirmed a significantly increased risk of invasive candidiasis infection with lower GNRI scores. Specifically, the following associations were observed: GNRI ≤ 98 (HR: 1.83, 95% CI: 1.23–2.72, P = 0.003), GNRI ≤ 92 (HR: 1.68, 95% CI: 1.17–2.4, P = 0.005), 82 ≤ GNRI ≤ 92 (HR: 1.63, 95% CI: 1.01–2.64, P = 0.046), GNRI ≤ 81 (HR: 2.31, 95% CI: 1.44–3.69, P < 0.001).

**Conclusions:**

Lower GNRI score was significantly associated with an increased risk of invasive candidiasis in elderly patients in ICU. Further research is needed to validate whether improving nutrition can prevent invasive candidiasis.

**Supplementary Information:**

The online version contains supplementary material available at 10.1186/s12879-023-08512-5.

## Background

Invasive candidiasis is the most common hospital-required fungal infection among patients in intensive care units (ICUs) [1]. The crude incidence of invasive candidiasis remains high at 13.3–26.2 per 100,000 person-years in the US due to the limited sensitivity of blood culture, difficulty in sampling deep tissues, and lengthy culturing time [2, 3]. As a commensal yeast, Candida *spp.* colonizes the skin and the intestines of healthy individuals without causing harm. Studies have shown that up to 60% of healthy individuals may carry Candida spp [4, 5]. However, candida spp. can translocate into the bloodstream or deep tissues, potentially causing disseminated infections in individuals with compromised local or systemic immunity. Invasive candidiasis is often accompanied by multiple organ dysfunction, including cardiac, hepatic, splenic, and renal systems, leading to severe systemic infections and sepsis [6].

Effective clinical interventions, such as source control and early administration of systemic anti-fungal medications, can improve prognostic outcomes [2, 7]. Despite the availability of potent antifungal agents, such as echinocandin, azole, and amphotericin B (AmB) in most ICUs, the crude mortality of invasive candidiasis is approximately 40% [4]. Thus, identifying and mitigating risk factors for invasive candidiasis are crucial to improve clinical outcomes for high-risk patients. Previous studies have identified various risk factors for invasive candidiasis, including age, diabetes, gastrointestinal perforation, sepsis, dialysis, broad-spectrum antibiotics use, immunosuppression, and total parenteral nutrition [8–10].

Nutritional support has become essential to manage intensive care patients in recent decades. However, 38–78% of ICU patients, especially those who are elderly, still experienced varying degrees of malnutrition [11]. Clinicians and researchers are increasingly focusing on exploring the relationship between malnutrition and the risk of infection [12, 13]. Malnutrition can dampen immune system, increase the risk of infection and in-hospital mortality in ICU patients [14, 15]. Moreover, two independent studies have shown that malnutrition is independently associated with the mortality of patients with invasive candidiasis [16, 17].

The Geriatric Nutritional Risk Index (GNRI) score is commonly used for evaluating the nutritional status of elderly patients. The score calculation is based on height, weight, and serum albumin, which are easily accessible indices [18]. Studies have demonstrated that GNRI is significantly associated with post-stroke cognitive outcomes, arrhythmia recurrence, heart failure, and frailty [19–22]. However, few studies have investigated the impact of GNRI on invasive candidiasis. In the study, we aim to investigate the association between GNRI and the risk of invasive candidiasis.

## Methods

### Data source and study population

Data were extracted from the Medical Information Mart for Intensive Care (MIMIC) IV and MIMIC-III Clinical Database CareVue subset (MIMIC-III_cv) databases. These databases contain hospitalization information ICU of the Beth Israel Deaconess Medical Center in the USA from 2008 to 2019, and from 2001 to 2008, separately. The data extraction process was carried out with official access permission and in compliance with all legislations and restrictions. All extracted records were deidentified to ensure that no individual patient-specific information could be identified or disclosed from the extracted datasets.

The inclusion criteria were as follows: (1) Age ≥ 65 years; (2) length of ICU hospitalization at least 24 h; and (3) availability of records containing information for GNRI calculation at admission. Patients without specific records required for GNRI calculation or those administered antifungal agents or diagnosed with invasive candidiasis before or within 48 h of ICU admission were excluded.

### Variables

All the following variables were considered as covariates: age, sex, service units (medical, surgical, trauma surgical, cardiac or cardiac surgical), the Sequential Organ Failure Assessment (SOFA) score, Simplified Acute Physiology Score II (SAPS II), mechanical ventilation and vasopressor use during the first 24 h of ICU admission. The comorbidities included congestive heart failure, renal diseases, liver diseases, chronic obstructive pulmonary diseases, and immunosuppression. Lymphoma, acquired immune deficiency syndrome, solid metastatic tumor, malignant tumor, autoimmune diseases, chemotherapy, or use of immunosuppressant were defined as immunosuppression status. Vital signs included heart rate, temperature, and mean arterial pressure. Laboratory measurements obtained in the first 24 h of ICU admission were included as follows: white blood cells, platelets, hemoglobin, serum creatinine, blood urea nitrogen, sodium, chloride, and bicarbonate. For measurements recorded more than once in the first 24 h, only those considered representing the most severe conditions were preserved in the data set.

According to the previous literature, we also included other risk factors for invasive candidiasis such as broad-spectrum antibiotic use, intravenous or oral corticosteroid use, and undergoing abdominal surgery before or within 48 h of ICU admission. Vancomycin, linezolid, carbapenems, quinolones, piperacillin/tazobactam, third or fourth generation cephalosporins, clarithromycin, clindamycin, doxycycline, and azithromycin were considered as broad-spectrum antibiotics. Corticosteroids included hydrocortisone, cortisone, prednisone, and dexamethasone. All related diseases were identified using the International Classification of Diseases, Ninth Revision (ICD-9), combined with Tenth Revision (ICD-10) diagnosis codes.

### Calculation of GNRI

The GNRI was calculated using the following formula in the study of Olivier Bouillanne et al[18], where ALB represents the serum albumin level (g/L) measured within 48 h before or after ICU admission because albumin levels were not measured on the admission day for a large proportion of patients. Weight was measured in kilograms and height was measured in meters.

$$GNRI=1.489\times ALB+41.7\times \left(\frac{Weight}{22\times {Height}^{2}}\right)$$

### Outcome

The primary outcome was invasive candidiasis, including bloodstream infection or deep-seated infections (intra-abdominal abscess, peritonitis, or osteomyelitis, with or without bloodstream infection) [10]. Invasive candidiasis was defined in the study as the detection of Candida spp. in blood, peritoneal fluid, or other sterile sites from the second day following admission to the ICU until discharge. However, samples such as urine, sputum, or bronchial washing were not considered as sources of invasive candidiasis.

### Sensitivity analysis

In the sensitivity analysis, we categorized the GNRI variable based on previous literature [18]. First, patients were divided into those with normal nutritional status (GNRI > 98) or malnutrition (GNRI ≤ 98). Among those with malnutrition, we further divided them into mild malnutrition group (GNRI: 92–98), moderate malnutrition group (GNRI: 82–92), and severe malnutrition group (GNRI ≤ 81). Alternatively, we also divided the entire cohort into those with normal nutritional status or mild malnutrition (GNRI > 92) and those with moderate or severe malnutritional (GNRI ≤ 92) using a cut-off GNRI value of 92.

### Statistical analysis

Categorical variables were described as frequency and percentage, and the chi-square test was used to assess the significance of differences between groups. Continuous variables that followed a normal distribution were presented as mean ± standard deviation, while those that did not follow a normal distribution were presented as median and quartiles. Independent sample t-tests or Mann-Whitney U tests were used to determine the significance of differences between the two groups. Initially, univariable Cox regression analysis was conducted to assess the variables significantly associated with the outcome. Subsequently, the variables found to be significant were included in a multivariable Cox regression model to evaluate independent risk factors for fungal infection. To further balance the potential confounding factors of multiple variables, propensity scores were calculated using a logistic regression model for variables that showed significant differences between the two groups. A 1:4 propensity score matched (PSM) patient cohort was then constructed, with a caliper value set at 0.03. All statistical analyses were performed using R software (version 4.1.3), and a significance level of P < 0.05 was considered statistically significant.

## Results

### Baseline characteristics

**Fig. 1:**
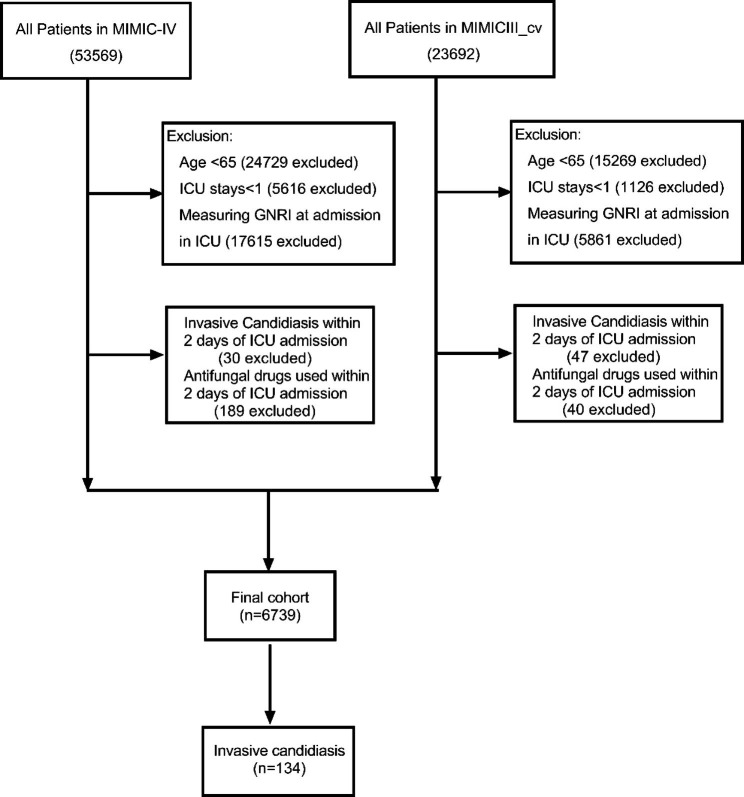
Flow chart of the cohort

A total of 6739 patients were included in the cohort from the MIMIC-IV database and MIMIC-III Clinical Database CareVue subset, among which 134 were diagnosed with invasive candidiasis during their ICU hospitalization (Fig. 1). For patients who were diagnosed with invasive candidiasis the median age was 75.12 [IQR: 70.14, 80.23] years and 55 patients (41.0%) were female. Among the 134 patients diagnosed with invasive candidiasis, there were various Candida species identified. Specifically, 94 had Candida albicans, 29 had Candida *glabrata*, 8 had Candida *parapsilosis*, 4 had Candida *tropicalis*, 2 had Candida *krusei*, 4 had Candida *lusitaniae*, 2 had Candida *dubliniensis*, and 1 patient had an unspecified Candida species. In addition, there were several cases of coinfection identified: coinfection of Candida *albicans* and Candida *parapsilosis* in 2 cases, coinfection of Candida *albicans* and Candida *krusei* in 1 case and coinfection of Candida *glabrata* and Candida *albicans* in 6 cases, *Candida albicans and Candida dubliniensis* coinfection were detected in 1 case. The specimens were obtained from blood culture (63 cases), swab (48 cases), body fluid (31 cases) and tissue (14 cases). The baseline characteristics of patients within each cohort were in Table 1.

**Table 1:** Baseline characteristics of patients with and without invasive candidiasis

Variables	Invasive candidiasis		
	No	Yes	P-value
Number of patients	6605	134	
GNRI	99.36 [87.98-110.45]	88.67 [79.26–98.27]	< 0.001
GNRI ≤ 98	3064 (46.4)	99 (73.9)	< 0.001
GNRI ≤ 92	2180 (33.0)	78 (58.2)	< 0.001
GNRI Category			< 0.001
GNRI > 98	3541 (53.6)	35 (26.1)	
92 < GNRI ≤ 98	884 (13.4)	21 (15.7)	
82 ≤ GNRI ≤ 92	1236 (18.7)	33 (24.6)	
GNRI ≤ 81	944 (14.3)	45 (33.6)	
Dialysis (%)	264 (4.0)	22 (16.4)	< 0.001
CVC (%)	4868 (73.7)	112 (83.6)	0.013
Service units (%)			< 0.001
MICU	2113 (32.0)	63 (47.0)	
SICU/TSICU	1286 (19.5)	38 (28.4)	
CCU/CSRU	3206 (48.5)	33 (24.6)	
Age	76.67 [70.86, 82.96]	75.12 [70.14, 80.23]	0.012
Female (%)	2902 (43.9)	55 (41.0)	0.562
SOFA	6 [4, 9]	10 [6, 13]	< 0.001
SAPSII	41[33, 51]	44 [36, 56]	0.004
CHF (%)	2764 (41.8)	54 (40.3)	0.786
Renal diseases (%)	1365 (20.7)	20 (14.9)	0.128
Liver diseases (%)	315 (4.8)	12 (9.0)	0.042
COPD (%)	1220 (18.5)	26 (19.4)	0.871
Diabetes mellitus (%)	2155 (32.6)	37 (27.6)	0.257
Immunosuppression (%)	154 (2.3)	3 (2.2)	1
Corticosteroids use (%)	639 (9.7)	22 (16.4)	0.014
Antibiotics (%)	3815 (57.8)	88 (65.7)	0.08
Abdominal operation (%)	480 (7.3)	23 (17.2)	< 0.001
Mechanical ventilation (%)	3156 (47.8)	94 (70.1)	< 0.001
Vasopressor (%)	1048 (15.9)	48 (35.8)	< 0.001
Temperature (℃)	37.00 [35.90, 37.61]	37.25 [35.89, 38.26]	0.014
Heart Rate (bpm)	88.00 [68.00, 116.00]	114.50 [69.00, 128.75]	< 0.001
MAP (mmHg)	69.00 [62.33, 104.00]	69.00 [49.00, 80.31]	0.458
WBC (×10^9^/L)	13.50 [9.80, 18.40]	15.36 [10.40, 20.17]	0.086
Hemoglobin (g/dL)	9.50 [8.10, 10.90]	8.90 [7.53, 10.40]	0.003
Platelets (×10^9^/L)	157.00 [111.00, 219.00]	139.50 [88.00, 217.00]	0.062
Sodium(mmol/L)	140.00 [137.00, 143.00]	141.00 [138.00, 144.75]	< 0.001
Chloride (mmol/L)	103.00 [100.00, 107.00]	103.00 [99.00, 108.00]	0.406
Bicarbonate (mmol/L)	22.00 [20.00, 26.00]	20.00 [16.00, 23.00]	< 0.001
BUN (mg/dL)	25.00 [17.00, 32.00]	27.50 [25.00, 41.00]	< 0.001
Creatinine (mg/dL)	1.20 [0.90, 1.80]	1.60 [1.10, 2.48]	< 0.001

Categorical variables were labeled ‘(%)’, which are presented as counts (percentages), and were compared through chi-squared tests. Continuous variables were presented as mean (standard deviation) and were compared through t-tests

Abbreviations: bpm: beats per minute; BUN: Blood urea nitrogen; CCU: Coronary care unit; CHF: Congestive heart failure; COPD: Chronic obstructive pulmonary disease; CSRU: Cardiac surgery recovery uni﻿t; ﻿C; VC: Central venous catheter; GNRI: Geriatric Nutritional Risk Index; MAP: Mean arterial pressure; MICU: Medical intensive care unit; SAPSII: Simplified Acute Physiology Score II; SICU: Surgical intensive care unit; SOFA: Sequential Organ Failure Assessment; TSICU: Trauma surgical intensive care unit; WBC: White blood cell

### The risk factors for invasive candidiasis

We performed collinearity tests on the data to ensure that there was no significant collinearity among the variables (Figure S1). The assumption of proportional hazards for the Cox regression model was met (P = 0.53), and the results of the nonlinearity test indicated a linear relationship between GNRI and the outcome (P for Nonlinear = 0.58). The results of the univariate Cox regression analysis are presented in Table S1, highlighting several factors associated with an increased risk of invasive candidiasis, such as dialysis (HR: 1.9, 95%CI: 1.2–3.1, P < 0.001), abdominal operation (HR: 1.8, 95%CI: 1.2–2.9, P < 0.001), higher heart rate (HR: 1, 95%CI: 1–1, P = 0.011), and higher BUN level (HR: 1, 95%CI: 1–1, P < 0.001). Moreover, as GNRI increased, the risk of infection decreased (HR: 0.98, 95%CI: 0.97–0.99, P < 0.001). As shown in Table 2, GNRI was identified as an independent predictor of invasive candidiasis (HR: 0.98, 95% CI: 0.97–0.99, P = 0.002).

**Table 2:** Comprehensive Results of Multivariable Cox Regression, Propensity Score Matching, and Sensitivity Analysis

	Univariable Cox regression		Multivariable Cox regression		Propensity score matching cohort	
	HR (95%CI)	P-value	HR (95%CI)	P-value	HR (95%CI)	P-value
GNRI	0.98 (0.97–0.99)	< 0.001	0.98 (0.97–0.99)	0.002	0.99 (0.98-1)	0.028
GNRI Category						
GNRI > 98	Ref	Ref	Ref	Ref	Ref	Ref
GNRI ≤ 98	2.2 (1.5–3.3)	< 0.001	1.83 (1.23–2.72)	0.003	1.7 (1.2–2.6)	0.005
GNRI Category						
GNRI > 92	Ref	Ref	Ref	Ref	Ref	Ref
GNRI ≤ 92	2 (1.4–2.8)	< 0.001	1.68 (1.17–2.4)	0.005	1.6 (1.1–2.3)	0.01
GNRI Category						
GNRI > 98	Ref	Ref	Ref	Ref	Ref	Ref
92 < GNRI ≤ 98	1.8 (1.04–3.12)	0.037	1.55 (0.89–2.71)	0.12	1.51 (0.87–2.62)	0.14
82 ≤ GNRI ≤ 92	1.88 (1.17–3.03)	0.009	1.63 (1.01–2.64)	0.046	1.82 (1.12–2.94)	0.015
GNRI ≤ 81	2.89 (1.85–4.5)	< 0.001	2.31 (1.44–3.69)	< 0.001	1.8 (1.15–2.81)	0.01

### Comparison of Propensity score-matched patient cohort

As shown in Table S2, a total of 132 cases (98.5%) with invasive candidiasis were matched with 527 controls in a 1:4 ratio. Apart from GNRI, there were no significant differences observed between the two groups for other variables. As presented in Table 2, GNRI remained significantly associated with invasive candidiasis in PSM cohort (HR: 0.99, 95%CI: 0.98-1, P = 0.028).

### Sensitivity analysis

We conducted a sensitivity analysis to categorize GNRI into three distinct groups. As shown in Table 2, compared to individuals with normal nutritional status (GNRI > 98), those with malnutrition (GNRI ≤ 98) had a significantly increased risk of invasive candidiasis in both the multivariable Cox regression model (HR: 1.83, 95%CI: 1.23–2.72, P = 0.003) and the PSM cohort (HR: 1.7, 95%CI: 1.2–2.6, P = 0.005). Furthermore, when individuals with normal nutritional status or mild malnutrition (GNRI > 92) were compared to those with moderate or severe malnutrition (GNRI ≤ 92), a higher risk of invasive candidiasis was observed in both the multivariable Cox regression model (HR: 1.68, 95% CI: 1.17–2.4, P = 0.005) and the PSM model (HR: 1.6, 95% CI: 1.1–2.3, P = 0.01).

Additionally, patients with moderate malnutrition (GNRI: 82–92) and severe malnutrition (GNRI ≤ 81) had increased risk of invasive candidiasis in the multivariable Cox regression model (HR: 1.63, 95%CI: 1.01–2.64, P = 0.046; HR: 2.31, 95%CI: 1.44–3.69, P < 0.001) and PSM model (HR: 1.82, 95%CI: 1.12–2.94, P = 0.015; HR: 1.8, 95%CI: 1.15–2.81, P = 0.01) compared to individuals with normal nutritional status (GNRI > 98).

## Discussion

In this study, we investigated the association between GNRI score and the risk of invasive candidiasis in elderly patients in the ICU. To our knowledge, this is the first study to investigate the association between GNRI score and the risk of invasive candidiasis. Our findings indicate that a lower GNRI score was significantly associated with an increased risk of invasive candidiasis. This suggests that nutritional status plays a crucial role in the susceptibility to this infection among elderly ICU patients.

Approximately 50% of hospitalized patients admitted to internal medicine units have hypoalbuminemia [23], which can exacerbate inflammation, decrease synthetic activities, and accelerate catabolism [24]. Several studies substantiated that hypoalbuminemia was independently associated with an increased risk of infection [25, 26]. The GNRI score can provide a more accurate evaluation of an individual’s nutritional status than simply using serum albumin levels and BMI as separate indicators. Yuta et al. [27] reported that a lower GNRI score was independently associated with an increased risk of infection-related mortality in patients receiving hemodialysis. Poor nutritional status indicated by a lower GNRI score was associated with an increased risk of surgical site infection after pancreatoduodenectomy and soft tissue sarcoma resection. Perioperative nutritional intervention effectively reduced the infection risk [28, 29]. Likewise, we reported a significant association between a lower GNRI score and a higher risk of invasive candidiasis infection. Although the exact mechanisms underlying this association remain unclear, impaired immune function may be partially responsible for the observed association [14]. It is common to use GNRI as a categorical variable in clinical settings instead of continuous variables [18, 30–32]. While GNRI is commonly used as a categorical variable in clinical settings for simplicity, our study showed that transforming GNRI, a continuous variable, into a categorical variable did not improve the predictive performance for the risk of invasive candidiasis.

It is important to note that our study focused on elderly patients in the ICU, as this population is particularly vulnerable to infections due to their compromised immune function and higher prevalence of malnutrition [33]. However, there are several limitations in this study. First, some patients were excluded from the study because of missing critical information due to the nature of retrospective study design. Furthermore, certain potential risk factors that could impact the incidence of invasive candidiasis, such as total parenteral nutrition, were not taken into consideration in this study due to unavailability in the database. Moreover, this study was unable to determine the effect of nutritional intervention, as indicated by improved GNRI scores, on the likelihood of invasive candidiasis in critically ill older adults as an observational study. Therefore, future prospective studies are in need to investigate the effect. Finally, the relationship between GNRI and invasive candidiasis may also be applicable to other patient populations. Future studies should aim to investigate this association in different clinical settings and patient cohorts to confirm the generalizability of our findings.

## Conclusions

In conclusion, this study observed that a lower GNRI score was associated with a significantly increased risk of invasive candidiasis in critically ill older adults. These findings highlight the importance of nutritional assessment and interventions in this vulnerable population. Further research is needed to validate our findings in larger and more diverse cohorts, explore potential preventive strategies, and elucidate the underlying mechanisms.

### Electronic supplementary material

Below is the link to the electronic supplementary material.

Supplementary Material 1

## Data Availability

The data were available on the MIMIC-IV website at https://mimic.physionet.org/. The datasets are available from the Massachusetts Institute of Technology (MIT) and Beth Israel Deaconess Medical Center (BIDMC) upon reasonable request according to the instructions for getting access to MIMIC-IV. The data in this article can be reasonably applied to the corresponding author (Prof. Liang Peng).

## Abbreviations

Bpm: beats per minute
BUN: Blood urea nitrogen
CCU: Coronary care unit
CHF: Congestive heart failure
COPD: Chronic obstructive pulmonary disease
CSRU: Cardiac surgery recovery unit
CVC: Central venous catheter
GNRI: Geriatric Nutritional Risk Index
MAP: Mean arterial pressure
MICU: Medical intensive care unit
SAPS II: Simplified Acute Physiology Score II
SICU: Surgical intensive care unit
SOFA: Sequential Organ Failure Assessment
TSICU: Trauma surgical intensive care unit
WBC: White blood cell
CI: Confidence interval
