# Supplementary material for: The association between Geriatric Nutritional Risk Index and the risk of Invasive Candidiasis in critically ill older adults
Source: BMC Infect Dis. 2023 Aug 14;23:530. doi: 10.1186/s12879-023-08512-5 (PMC10426167; doi:10.1186/s12879-023-08512-5)
Supplement: Supplementary file 1 — Supplementary Material 1 [file 12879_2023_8512_MOESM1_ESM.docx]

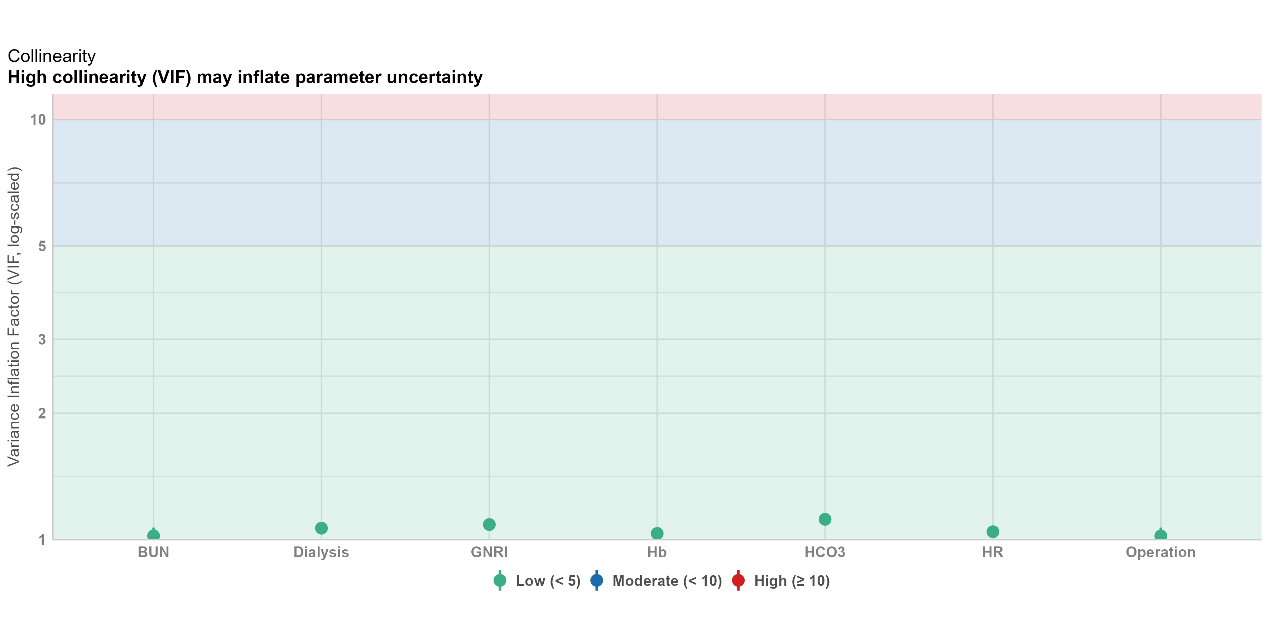


**Supplementary Figure 1**

Multicollinearity and variance inflation factor in the regression model. The variance inflation factor of each covariate not exceeding 2 means no obvious multicollinearity requiring correction.

**Supplementary Table 1 Univariates analysis of the cohort**

| Covariate | HR (95%CI) | P-value |
| --- | --- | --- |
| GNRI | 0.98 (0.97-0.99) | <0.001 |
| Dialysis (%) | 1.9 (1.2-3.1) | <0.001 |
| CVC (%) | 1.1 (0.68-1.7) | 0.77 |
| Service units (%) |  |  |
| MICU | Ref |  |
| SICU/TSICU | 0.69(0.46-1.03) | 0.07 |
| CCU/CSRU | 0.47(0.3-0.71) | <0.001 |
| Age | 1 (0.99-1) | 0.77 |
| Female (%) | 0.91 (0.64-1.3) | 0.57 |
| SOFA | 1 (0.99-1.1) | 0.15 |
| SAPSII | 1 (0.99-1) | 0.92 |
| CHF (%) | 0.81 (0.57-1.1) | 0.24 |
| Renal diseases (%) | 0.65 (0.4-1.1) | 0.078 |
| Liver diseases (%) | 1.5 (0.84-2.7) | 0.17 |
| COPD (%) | 0.84 (0.55-1.3) | 0.44 |
| Diabetes mellitus (%) | 0.79 (0.54-1.2) | 0.23 |
| Immunosuppression (%) | 0.36 (0.088-1.5) | 0.15 |
| Corticosteroids use (%) | 1.2 (0.74-1.9) | 0.5 |
| Abdominal operation (%) | 1.8 (1.2-2.9) | <0.001 |
| Mechanical ventilation (%) | 1.4 (0.95-2) | 0.088 |
| Antibiotics (%) | 1.2 (0.83-1.7) | 0.34 |
| Vasopressor (%) | 1.4 (0.97-2) | 0.077 |
| Temperature (℃) | 1.1 (0.98-1.2) | 0.11 |
| Heart Rate (bpm) | 1 (1-1) | 0.011 |
| MAP (mmHg) | 1 (0.99-1) | 0.2 |
| WBC (×10^9^/L) | 1 (0.99-1) | 0.65 |
| Hemoglobin (g/dL) | 0.91 (0.84-0.99) | 0.022 |
| Platelets (×10^9^/L) | 1 (1-1) | 0.77 |
| Sodium(mmol/L) | 1 (0.99-1.1) | 0.14 |
| Chloride (mmol/L) | 1 (1-1.1) | 0.07 |
| Bicarbonate (mmol/L) | 0.94 (0.91-0.97) | <0.001 |
| BUN (mg/dL) | 1 (1-1) | <0.001 |
| Creatinine (mg/dL) | 1.1 (0.98-1.2) | 0.12 |

Abbreviations: BUN: Blood urea nitrogen; CCU: Coronary care unit; CHF: Congestive heart failure; COPD: Chronic obstructive pulmonary disease; CSRU: Cardiac surgery recovery unit; CVC: Central venous catheter; GNRI: Geriatric Nutritional Risk Index; MAP: Mean arterial pressure; MICU: Medical intensive care unit; SAPSII: Simplified Acute Physiology Score II; SICU: Surgical intensive care unit; SOFA: Sequential Organ Failure Assessment; TSICU: Trauma surgical intensive care unit; WBC: White blood cell.

**Supplementary Table 2: Baseline characteristics of the propensity score-matched patient cohort.**

| **Features** | **Invasive candidiasis** | |  |
| --- | --- | --- | --- |
|  | **No** | **Yes** | **P-value** |
| n | 527 | 132 |  |
| GNRI | 94.61 [84.10,105.66] | 89.09 [79.41,98.42] | 0.002 |
| GNRI≤98 | 310 (58.8) | 97 (73.5) | 0.003 |
| GNRI≤92 | 230 (43.6) | 76 (57.6) | 0.006 |
| GNRI≤80.79 | 103 (19.5) | 42 (31.8) | 0.003 |
| GNRI Category |  |  | 0.008 |
| GNRI>98 | 217 (41.2) | 35 (26.5) |  |
| 92<GNRI≤98 | 80 (15.2) | 21 (15.9) |  |
| 82≤GNRI≤92 | 114 (21.6) | 32 (24.2) |  |
| GNRI≤81 | 116 (22.0) | 44 (33.3) |  |
| Dialysis (%) | 83 (15.7) | 21 (15.9) | 1 |
| CVC (%) | 444 (84.3) | 110 (83.3) | 0.901 |
| Service units (%) |  |  | 0.99 |
| MICU | 246 (46.7) | 62 (47.0) |  |
| SICU/TSICU | 151 (28.7) | 37 (28.0) |  |
| CCU/CSRU | 130 (24.7) | 33 (25.0) |  |
| Age | 76.33 [70.95,82.57] | 76.41 [70.37,81.74] | 0.11 |
| Female (%) | 239 (44.6) | 231 (43.8) | 0.611 |
| SOFA | 10 [7,13] | 10 [6,13] | 0.736 |
| SAPSII | 44 [35,57] | 44 [36,55] | 0.825 |
| CHF (%) | 231(43.8) | 53 (40.2) | 0.506 |
| Renal diseases (%) | 126(23.9) | 20 (15.2) | 0.04 |
| Liver diseases (%) | 46(8.7) | 12 (9.1) | 1 |
| COPD (%) | 102(19.4) | 26 (19.7) | 1 |
| Diabetes mellitus (%) | 179(34.0) | 36 (27.3) | 0.173 |
| Immunosuppression (%) | 15(2.8) | 3 (2.3) | 0.95 |
| Corticosteroids use (%) | 80(15.2) | 21 (15.9) | 0.942 |
| Antibiotics (%) | 360 (68.3) | 87 (65.9) | 0.671 |
| Abdominal operation (%) | 80(15.2) | 22 (16.7) | 0.774 |
| Mechanical ventilation (%) | 367(69.6) | 92 (69.7) | 1 |
| Vasopressor (%) | 187(35.5) | 46 (34.8) | 0.972 |
| Temperature (℃) | 37.17 [35.90,37.94] | 37.22 [35.89,38.18] | 0.456 |
| Heart Rate (bpm) | 112.00 [69.00,127.00] | 114.00 [69.00,127.25] | 0.858 |
| MAP (mmHg) | 69.00 [49.00,109.33] | 69.00 [49.00,80.31] | 0.622 |
| WBC (×10^9^/L) | 15.10 [10.35,21.95] | 15.31 [10.33,20.12] | 0.438 |
| Hemoglobin (g/dL) | 8.90 [7.50,10.40] | 8.90 [7.50,10.40] | 0.781 |
| Platelets (×10^9^/L) | 145.00 [91.00,218.00] | 140.00 [90.25,220.50] | 0.945 |
| Sodium(mmol/L) | 141.00 [138.00,144.00] | 141.00 [138.00,144.25] | 0.612 |
| Chloride (mmol/L) | 104.00 [100.00,107.00] | 103.00 [99.00,108.00] | 0.645 |
| Bicarbonate (mmol/L) | 20.00 [16.50,23.00] | 20.00 [16.00,23.00] | 0.595 |
| BUN (mg/dL) | 26.07 [22.00,40.00] | 27.00 [24.50,40.25] | 0.628 |
| Creatinine (mg/dL) | 1.60 [1.10,2.60] | 1.60 [1.10,2.45] | 0.782 |

Categorical variables were labeled ‘(%)’, which are presented as counts (percentages), and were compared through chi-squared tests. Continuous variables were presented as mean (standard deviation) and were compared through t-tests.

Abbreviations: bpm: beats per minute; BUN: Blood urea nitrogen; CCU: Coronary care unit; CHF: Congestive heart failure; COPD: Chronic obstructive pulmonary disease; CSRU: Cardiac surgery recovery unit; CVC: Central venous catheter; GNRI: Geriatric Nutritional Risk Index; MAP: Mean arterial pressure; MICU: Medical intensive care unit; SAPSII: Simplified Acute Physiology ScoreII; SICU: Surgical intensive care unit; SOFA: Sequential Organ Failure Assessment; TSICU: Trauma surgical intensive care unit; WBC: White blood cell.
